# Once‐ and twice‐daily heat acclimation confer similar heat adaptations, inflammatory responses and exercise tolerance improvements

**DOI:** 10.14814/phy2.13936

**Published:** 2018-12-21

**Authors:** Ashley G. B. Willmott, Mark Hayes, Carl A. James, Jeanne Dekerle, Oliver R. Gibson, Neil S. Maxwell

**Affiliations:** ^1^ Environmental Extremes Laboratory University of Brighton Brighton Eastbourne United Kingdom; ^2^ Institut Sukan Negara (National Sports Institute) National Sports Complex Kuala Lumpur Malaysia; ^3^ Centre for Human Performance, Exercise and Rehabilitation (CHPER) Brunel University London Uxbridge United Kingdom

**Keywords:** heat acclimatization, heat adaptation, immune, performance, thermoregulation, training

## Abstract

This experiment aimed to investigate the efficacy of twice‐daily, nonconsecutive heat acclimation (TDHA) in comparison to once‐daily heat acclimation (ODHA) and work matched once‐ or twice‐daily temperate exercise (ODTEMP, TDTEMP) for inducing heat adaptations, improved exercise tolerance, and cytokine (immune) responses. Forty males, matched biophysically and for aerobic capacity, were assigned to ODHA, TDHA, ODTEMP, or TDTEMP. Participants completed a cycling‐graded exercise test, heat acclimation state test, and a time to task failure (TTTF) at 80% peak power output in temperate (TTTF_TEMP_: 22°C/40% RH) and hot conditions (TTTF_HOT_: 38°C/20% RH), before and after 10‐sessions (60 min of cycling at ~2 W·kg^−1^) in 45°C/20% RH (ODHA and TDHA) or 22°C/40% RH (ODTEMP or TDTEMP). Plasma IL‐6, TNF‐*α*, and cortisol were measured pre‐ and postsessions 1, 5, and 10. ODHA and TDHA induced equivalent heat adaptations (*P* < 0.05) (resting rectal temperature [−0.28 ± 0.22, −0.28 ± 0.19°C], heart rate [−10 ± 3, −10 ± 4 b·min^−1^], and plasma volume expansion [+10.1 ± 5.6, +8.5 ± 3.1%]) and improved heat acclimation state (sweat set point [−0.22 ± 0.18, −0.22 ± 0.14°C] and gain [+0.14 ± 0.10, +0.15 ± 0.07 g·sec^−1^·°C^−1^]). TTTF_HOT_ increased (*P* < 0.001) following ODHA (+25 ± 4%) and TDHA (+24 ± 10%), but not ODTEMP (+5 ± 14%) or TDTEMP (+5 ± 17%). TTTF_TEMP_ did not improve (*P* > 0.05) following ODHA (+14 ± 4%), TDHA (14 ± 8%), ODTEMP (9 ± 10%) or TDTEMP (8 ± 13%). Acute (*P* < 0.05) but no chronic (*P* > 0.05) increases were observed in IL‐6, TNF‐*α*, or cortisol during ODHA and TDHA, or ODTEMP and TDTEMP. Once‐ and twice‐daily heat acclimation conferred similar magnitudes of heat adaptation and exercise tolerance improvements, without differentially altering immune function, thus nonconsecutive TDHA provides an effective, logistically flexible method of HA, benefitting individuals preparing for exercise‐heat stress.

## Introduction

Heat acclimation (HA) is an important preparation strategy preceding exercise‐heat stress (Périard et al. 2015; Racinais et al. 2015) to alleviate physiological strain (Nybo et al. 2014), attenuate heat related illness (HRI) (Yamazaki 2012), improve thermal perception (Gonzalez and Gagge 1976) and exercise tolerance in hot (Nielsen et al. 1993), and possibly temperate conditions (Lorenzo et al. 2010). A variety of HA strategies currently exist, predominantly differentiated by exercise‐heat stress volume, and/or intensity (Taylor 2014; Daanen et al. 2017). In this regard, HA may be applied within sporting and occupational settings (e.g., military), with current recommendations advocating the use of repeated, consecutive once‐daily exertional heat exposures for 60–100 min, utilizing an isothermic protocol (Racinais et al. 2015). In spite of multiple manipulations of volume/intensity, the optimal frequency for HA remains largely unknown (Tyler et al. 2016). Current recommendations for once‐daily exposures are implied more commonly than nonconsecutive [e.g., 10‐sessions in 21‐days (Gill and Sleivert 2001)] and twice‐daily exposures [e.g., 100 min vs. 2 × 50 min (Lind and Bass 1963)], due to the consistency of potentiating stimuli for adaptation, for example daily elevations in rectal [*T*_re_] and skin temperature alongside profuse sweating, which are required to evoke a multitude of physiological and perceptual adaptations (Sawka et al. 2011). From a practical perspective, implementing consecutive‐day protocols is challenging given access to hot‐humid conditions is not ubiquitous, and the need for daily exposures is likely to interrupt sport/occupational‐specific training, competition tapering and, or travel/recovery schedules. Medium‐ (MTHA: 10–14‐days) and long‐term (LTHA: >14‐days) protocols which maximize adaptations exacerbate these challenges, a factor which may provide some explanation as to why, in spite of clear recommendations from the scientific community, only ~15% of athletes undertook HA prior to competition in heat stress (Périard et al. 2017).

We have previously shown that four HA sessions, that is a short‐term HA (STHA) intervention (Willmott et al. 2016), administered over two consecutive days (i.e., twice‐daily HA [TDHA]), demonstrated comparable adaptations to four consecutive once‐daily HA (ODHA) sessions. However the magnitudes of adaptation using STHA are typically smaller than MTHA/LTHA interventions, thus the need to examine the efficacy of a twice‐daily approach over longer periods exists. Furthermore given challenges associated with consecutive day interventions, completing TDHA intermittently (e.g., over nonconsecutive days), over MTHA/LTHA timescales, may be desirable given an improved ability to integrate HA into complex training and travel schedules, potentially reducing disruption. For example, by administering the same number of HA sessions (i.e., the same dose) nonconsecutively, athletes may be afforded recovery days during HA or have the ability to perform specific training on non‐HA days. While hypothetically beneficial, investigations are needed to assess the efficacy of this strategy, particularly given different markers of heat adaptation have differing time courses for induction (Periard et al. 2016) and the associations between adaptation and performance enhancement are not ubiquitously reported. Previous research findings are equivocal, with suboptimal adaptations reported during nonconsecutive versus daily HA (Gill and Sleivert 2001), attributable to heat decay (Weller et al. 2007), and insufficient physiological stimulus (Barnett and Maughan 1993). Consequently, refining nonconsecutive protocols so that the timescale, protocol, and dose are in line with best practice recommendations, that is using an isothermic model of ~10‐sessions over 10–14‐days (Racinais et al. 2015) and thus, ensuring twice‐daily methods implement appropriate potentiating stimuli, may ameliorate current limitations and provide an alternative strategy for practitioners who pursue HA benefits but prioritize training quality and recovery schedules.

While acute exercise‐heat stress is unlikely to impair immune function (Walsh et al. 2011; Walsh and Oliver 2016), few studies have investigated immunological biomarkers during HA despite the potential for immunological perturbations to culminate in exacerbated inflammatory (e.g., interleukin‐6 [IL‐6] and tumor necrosis factor‐alpha [TNF‐*α*]) and stress responses (e.g., cortisol) (Willmott et al. 2017; Costello et al. 2018), potentially increasing HRI susceptibility (Leon and Helwig 2010), and diminishing the application and efficacy of HA (Pyne et al. 2014; Guy et al. 2016). Investigation of inflammatory responses to once‐daily isothermic HA reported few negative findings (Costello et al. 2018); however, the immune response to our proposed twice‐daily model of matched volume (dose), but altered frequency, remains unknown and maladaptation may be a concern. Therefore, investigation is required given the repeated exercise‐induced hyperthermia, coupled with shorter recovery time during the “heat days” of TDHA that may result in an overload of physiological strain, inducing residual stress between sessions (Ronsen et al. 2004).

This study investigated the efficacy of short‐ (i.e., 5‐sessions) and medium‐term (i.e., 10‐sessions) HA, using nonconsecutive TDHA and consecutive ODHA protocols, and compared these to temperate exercise groups (i.e., once‐daily: ODTEMP and nonconsecutive twice‐daily: TDTEMP) as frequency and duration matched exercise controls. Secondly, this study investigated exercise tolerance through the determinants of aerobic performance, and subsequent performance in both hot and temperate conditions between interventions. Finally, this study also investigated the inflammatory and stress responses during interventions to determine whether a compromised immune function was an artifact of the twice‐daily protocol. It was hypothesized that as the dose of HA was the same, TDHA would induce the same physiological and ergogenic benefits as ODHA, with both TDHA and ODHA superior to ODTEMP and TDTEMP. Given the alteration in frequency of the HA dose, it was hypothesized that the reduced duration between TDHA sessions would lead to undesirable inflammatory/stress responses in comparison to ODHA.

## Methods

### Participants and ethical approval

Forty moderately‐trained [performance level 3 (De Pauw et al. 2013)] males provided informed consent to participate in the experiment, which was approved by the University of Brighton Institution's Research Ethics and Governance Committee and conducted in accordance with Declaration of Helsinki (2013). Participants were matched for biophysical characteristics and aerobic capacity and assigned to: consecutive ODHA, nonconsecutive TDHA, consecutive ODTEMP, or nonconsecutive TDTEMP. No differences in participant characteristics were observed (*P* > 0.05 [Table 1]),

**Table 1:** Mean ± standard deviation (SD) participant characteristics

Group (*n* = 40)	Age (years)	Body mass (kg)	Height (m)	BMI (kg·m^2^)	BSA (m^2^)	Sum of skinfolds (mm)	Body fat (%)
ODHA (*n* = 10)	23 ± 6	77.2 ± 10.0	1.78 ± 0.08	24.4 ± 2.1	1.95 ± 0.16	34.5 ± 7.3	14.9 ± 2.7
TDHA (*n* = 10)	25 ± 7	75.3 ± 9.5	1.79 ± 0.04	23.4 ± 2.5	1.94 ± 0.13	33.4 ± 9.9	14.3 ± 3.7
ODTEMP (*n* = 10)	22 ± 1	77.3 ± 8.6	1.77 ± 0.04	25.5 ± 3.0	1.92 ± 0.10	35.7 ± 6.4	15.0 ± 1.7
TDTEMP (*n* = 10)	22 ± 1	75.2 ± 7.8	1.78 ± 0.07	23.8 ± 1.5	1.93 ± 0.14	33.8 ± 7.5	14.6 ± 2.9

BMI, body mass index; BSA, body surface area; ODHA, once‐daily heat acclimation; TDHA, twice‐daily heat acclimation; ODTEMP, once‐daily temperate exercise; TDTEMP, twice‐daily temperate exercise.

### Experimental design

**Figure 1:**
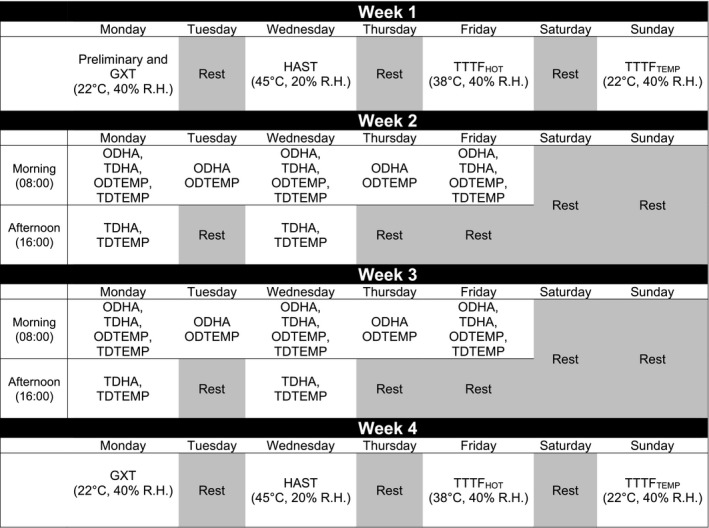
Schematic design of the study. Note HAST, TTTF_HOT_ and TTTF_TEMP_ performed in randomized order. GXT = graded exercise test, HAST = heat acclimation state test, TTTF = time to task failure in hot (_HOT_) or temperate (_TEMP_) conditions, ODHA = once‐daily heat acclimation, TDHA = twice‐daily, nonconsecutive day heat acclimation, ODTEMP = once‐daily temperate training, TDTEMP = twice‐daily, nonconsecutive day temperate training.

Prior to group allocation, participants completed four tests comprising: cycling graded exercise test (GXT), heat acclimation state test (HAST) and time to task failure test in hot (TTTF_HOT_) and temperate conditions (TTTF_TEMP_), in a semi‐randomized order, 48 h apart with the GXT completed first. Interventions consisted of, 60‐min exercise sessions performed in hot (45°C, 20% RH) or temperate conditions (22°C, 40% RH) over a 12‐day period. Post‐tests were repeated in the same order 48 h apart (Fig. 1). This study was completed during November–February, with trials occurring at the same time of day to minimize the effect of circadian variation on exercise tolerance (Drust et al. 2005) and thermoregulation (Waterhouse et al. 2005). Participants avoided alcohol and caffeine 12 h before experimentation, arrived in a euhydrated state (Sawka et al. 2007) and replicated food intake the day of the each exercise trial (Bailey et al. 2012).

### Determinants of aerobic performance – graded exercise test

Height (Detecto Scale Company, USA) and body mass (Adam Equipment Inc., USA) were measured, enabling the estimation of body surface area (BSA) (Du Bois and Du Bois 1916). Skinfold thickness was measured (Harpenden, Baty International, UK) across four sites (Durnin and Womersley 1974) to estimate body fat (%) (Siri 1956). The GXT was completed on an electronically braked stationary ergometer (SRM High performance model, Germany) within temperate conditions (22°C, 40% RH). Power output was initially set at 80 W and increased by 24 W every stage (3 min), with cadence maintained at 80 rev·min^−1^. Capillary blood lactate concentration ([La]_b_) was sampled within the final 30‐sec of each stage and analyzed immediately (2300 Plus, YSI, USA). Breath‐by‐breath metabolic gas data were continuously collected (Metalyzer 3B, Cortex, Germany). Lactate threshold (LT) was determined by an increase (>1 mmol·L^−1^) in [La]_b_ above resting level (Coyle 1999) and the test was terminated when the onset of blood lactate accumulation (OBLA) occurred (>4 mmol·L^−1^) (Winter et al. 2007). Gross mechanical efficiency (GME) was calculated from steady‐state oxygen consumption and respiratory exchange ratio (RER < 1.0) values collected during the final 30‐sec of each stage of the LT test (Garby and Astrup 1987). Following 15 min rest, participants performed a second test with an initial power output 48 W below OBLA that was increased by 20 W·min^−1^ until volitional exhaustion (Hayes et al. 2014). Peak oxygen uptake ($\dot{V}O_{2\mathrm{peak}}$) and power output (PPO) were determined as the highest average $\dot{V}O_{2}$ and power output during the final 30‐sec of each stage. Following 15 min rest, participants were familiarized to the TTTF at 80% of their PPO.

### Aerobic performance – time to task failure

TTTF_TEMP_ (22°C, 40% RH) and TTTF_HOT_ (38°C, 40% RH) were completed at 80% of PPO (McLellan et al. 1995) on a modified cycle ergometer (SRM crankset and wireless PowerControl meter on a Monark 874E, Sweden). Following a standardized warm‐up (2 min seated rest, 5 min at 90% of LT, 3 min rest, and then 3 min of unloaded pedaling at 80 rev·min^−1^), power output was increased to 80% PPO. HR, *T*_re_, and metabolic gas data were collected every minute and RPE was recorded at task failure (i.e., when cadence failed <77 rev·min^−1^ for >3‐sec following a warning). Power output, HR and time were obscured with only cadence displayed.

### Heat acclimation state test

HASTs were completed in hot‐dry conditions (45°C, 15% RH) within an environmental chamber (TISS, UK) on a cycle ergometer (Monark 620, Sweden). HASTs replicated an established protocol (Havenith and H 1986), but prescribed exercise intensities at given rates of ${\dot{H}}_{\mathrm{prod}}$ relative to body mass (3.0, 4.5 and 6.0 W·kg^−1^) (Willmott et al. 2015). Heat acclimation state was identified via sweat set point and sweat gain measures (Havenith and van Middendorp 1990). Metabolic energy expenditure was estimated from known values of $\dot{V}O_{2}$ and RER below LT during the GXT (Nishi 1981). ${\dot{H}}_{\mathrm{prod}}$ was subsequently calculated and associated exercise intensities prescribed during the HAST (Cramer and Jay 2014), which were recalculated postintervention.

### Heat acclimation and temperate exercise protocols

Participants completed ten 60‐min exercise sessions over 12 days. Once‐a‐day groups (ODHA, ODTEMP) exercised on days 1–5 and 8–12 at 08:00 h, whereas twice‐daily groups (TDHA, TDTEMP) exercised twice on days 1, 3, 8, and 10 at 08:00 h and 16:00 h, and then once on days 5 and 12 at 08:00 h (Fig. 1). Exercise commenced at 2.3 W·kg^−1^ (~65% $\dot{V}O_{2\mathrm{peak}}$) for 15 min at 80 rev·min^−1^, in line with recommended guidelines to rapidly attain the desired change in core temperature (Gibson et al. 2017b; James et al. 2017). Power output was subsequently altered depending on changes in *T*_re_ (∆*T*_re_) and perceived effort (Neal et al. 2015), to target a *T*_re_ of ≥38.5°C for the remainder of the session (Taylor 2014) (see Table 2 for actual training data). To amplify ∆*T*_re_, upper‐body sauna suits (Everlast, London, UK) (Willmott et al. 2018) were worn during the initial 15 min of exercise. This method has been applied prior to HA (Mee et al. 2018) to increase physiological strain without increasing exercise intensity or volume (Dawson 1994; Willmott et al. 2018). Physiological and perceptual measures were recorded at rest and every 5 min during exercise for all 10 sessions. During sessions 1, 5, and 10, fluid ingestion was prohibited for accurate estimation of sweat loss. Participants were permitted to drink at ad libitum during the remaining sessions (Neal et al. 2015). Euhydration was determined on arrival to each session by collection of mid flow urine; color <3 (U_col_), osmolality <700 mOsmol·kg^−1^ (U_osm_) (Osmocheck, Vitech Scientific Ltd., Japan) and specific gravity <1.020 (U_sg_) (hand refractometer, Atago, Japan) (Sawka et al. 2007). HR was manually recorded (Polar Electro, Oy, Finland) and *T*_re_ was continuously monitored using a thermistor probe (Henleys Medical Supplies, UK) self‐inserted 10 cm past the anal sphincter. Whole‐body sweat loss (WBSL) was estimated for each session from towel dried nude body mass differences pre‐ to postexercise. Sweat samples (~2 mL) were collected in a Tegaderm+Pad (3M™, USA) placed on the midpoint of the trapezius before being analyzed for sodium concentration ([Na^+^]) using a Sweat‐Chek™ (Eli Tech Group, Wescor Inc., USA) for sessions 1, 5, and 10.

**Table 2:** Mean ± SD changes (∆) in heat adaptations over days 1–5 (short‐term) and days 1–10 (medium‐term) and during the heat acclimation state pre‐ postintervention

Group	ODHA		TDHA		ODTEMP		TDTEMP	
Session	1–5	1–10	1–5	1–10	1–5	1–10	1–5	1–10
Heat adaptations								
∆Rest *T*_re_ (°C)	−0.18 ± 0.27*	−0.28 ± 0.22*, †, ‡, +	−0.22 ± 0.17*, †, ‡	−0.28 ± 0.19*, †, ‡, +	+0.03 ± 0.21	−0.10 ± 0.16	−0.04 ± 0.17	−0.11 ± 0.18
∆Rest HR (b·min^−1^)	−5 ± 1*	−10 ± 3* ^+^	−5 ± 5*	−10 ± 4*, +	−1 ± 1	−2 ± 1	+1 ± 3	−2 ± 6
∆PV (%)	+6.3 ± 4.0	+10.1 ± 5.6*, +	+5.4 ± 4.0	+8.5 ± 3.1*, +	+0.5 ± 2.8	+1.5 ± 3.5	+1.5 ± 3.4	+0.7 ± 4.1
∆WBSL (mL)	+230 ± 207*	+533 ± 261*, †, ‡, +	+178 ± 142*, †, ‡	+398 ± 97*, †, ‡, +	+83 ± 86	+81 ± 97	+48 ± 68	+90 ± 118
∆ [Na^+^] (mmol·L^−1^)	−13 ± 13*, †, ‡	−27 ± 19*, +, †, ‡	−7 ± 6	−14 ± 5*, +	−12 ± 12	−24 ± 20+	−6 ± 12	−11 ± 13+
∆RPE_peak_	−1 ± 1	−2 ± 1*, +	−1 ± 1	−2 ± 1*, +	−1 ± 1	−2 ± 1*, +	0 ± 2	0 ± 2
∆TSS_peak_	−0.3 ± 0.4	−0.7 ± 0.5*, +	−0.5 ± 0.5	−0.9 ± 0.5*, +	+0.4 ± 0.5	+0.2 ± 0.8	+0.1 ± 0.9	+0.1 ± 0.7
∆TC_peak_	−1 ± 1	−1 ± 1*	0 ± 1	−1 ± 1*, +	0 ± 0	0 ± 0	0 ± 0	0 ± 0
Heat acclimation state (1–10)								
∆Sweat set point (°C)	−0.22 ± 0.18*, †		−0.22 ± 0.14*, ‡, †		−0.14 ± 0.18*		−0.11 ± 0.10*	
∆Sweat gain (g·sec^−1^·°C^−1^)	+0.14 ± 0.10*		+0.15 ± 0.07*		+0.05 ± 0.07		+0.06 ± 0.06	
∆WBSL (mL)	+262 ± 180*		+278 ± 211*		+68 ± 118		+68 ± 112	
∆*T*_repeak_ (°C)	−0.25 ± 0.11*		−0.28 ± 0.11*		−0.15 ± 0.27		−0.08 ± 0.25	
∆HR_peak_ (b·min^−1^)	−13 ± 9*		−14 ± 10*		−4 ± 1		−2 ± 6	
∆RPE_peak_	−3 ± 2*		−3 ± 1*		−3 ± 2*		−2 ± 2*	
∆TSS_peak_	−0.7 ± 0.5		−0.6 ± 0.7		−0.4 ± 0.9		−0.3 ± 0.4	
∆TC_peak_	−1 ± 1*, ‡		−1 ± 1*, ‡		−1 ± 1*		0 ± 1	

ODHA, once‐daily heat acclimation; TDHA, twice‐daily heat acclimation; ODTEMP, once‐daily temperate exercise; TDTEMP, twice‐daily temperate exercise; *T*_re_, rectal temperature; HR, heart rate; PV, plasma volume; WBSL, whole‐body sweat loss; [Na^+^], sodium concentration; RPE, rating of perceived exertion; TSS, thermal sensation; TC, thermal comfort.

* Represents a significant (*P* < 0.05) within‐group difference.

† Represents a significant (*P* < 0.05) between‐group difference with ODTEMP.

‡ Represents a significant (*P* < 0.05) between‐group difference with TDTEMP.

+ Represents a significant difference (*P* < 0.05) between 1–5 and 1–10 adaptations.

### Phlebotomy and biochemistry

Following 10 min of seated rest immediately before and after sessions 1, 5, and 10, fingertip capillary blood (~200 *μ*L) was sampled for hemoglobin (HemoCue, Ltd., Sweden) and hematocrit (Hawksley and Sons Ltd., England) to estimate ΔPV (Dill and Costill 1974). A 10 mL venepuncture sample was also collected from the antecubital fossa, transferred into two 5 mL tubes (EDTA Sarstedt, Akteingesellscaft and Co, Germany), centrifuged (Eppendorf 5702 R Centrifuge, UK) for 10 min at 5000 rev·min^−1^, and then plasma stored at −86°C. Upon analysis, commercially available ELISA kits were used to measure IL‐6 and TNF‐*α* (*Ready Set Go!* ^*®*^, eBioscience, Affymetrix Inc., USA) and cortisol (Sigma‐Aldrich, USA) in duplicate and corrected for ∆PV.

### Perceptual measures

RPE (Borg 1982) from 6 (*No exertion*) to 20 (*Maximal Exertion*), thermal sensation scale [TSS (Toner et al. 1986) from 0 (*Very Very Cold*), 4 (*Neutral*) to 8 (*Very Very Hot*)] and thermal comfort [TC (Zhang et al. 2004) from 0 (*Very Comfortable*) to 5 (*Very Uncomfortable*)] were collected during exercise sessions every 5 min following familiarization.

### Data and statistical analyses

All data are reported as mean ± SD, with statistical significance set at *P* < 0.05. Data were assessed and conformed to normality and sphericity prior to further statistical analysis. Within‐group differences for pre‐intervention data sets were analyzed using a one‐way ANOVA. To assess intervention efficacy, physiological, performance and perceptual data were analyzed using a three‐way mixed design ANOVA (Time*Condition*Frequency), for time (pre‐ to postintervention), condition (HA and TEMP) and frequency (once‐ and twice‐daily exercise). Following a significant *F*‐value, follow up Bonferroni‐corrected post‐hoc comparisons were used. Predefined analytical limits to highlight meaningful heat adaptations were: ∆*T*_re_  > 0.20°C, ∆HR > 5 b·min^−1^ ∆WBSL > 200 mL, ∆PV > 5% and >1 in perceptual scales (RPE, TSS, and TC) (Willmott et al. 2017). Typical error of measurement (TEM) were used to determine meaningful differences for sweat set point (0.21°C), sweat gain (0.09 g·sec^−1^·°C^−1^), TTTF test (15%), $\dot{V}O_{2\mathrm{peak}}$ test (4.8%), IL‐6 (2 pg·mL^−1^), TNF‐*α* (1 pg·mL^−1^), and cortisol (57 nmol·L^−1^). Isotime data (i.e., task failure time‐point preintervention compared to the corresponding time‐point postintervention) was also analyzed.

## Results

### Heat adaptations

During both ODHA and TDHA interventions, resting *T*_re_, resting HR, and sweat [Na^+^] were reduced, while WBSL and PV were increased within sessions 5 (STHA) and 10 (MTHA) (*P* < 0.05) compared to session 1 (Table 2). The highest recorded perceptual measures (i.e., peak RPE, TSS, and TC) were also lower (*P* < 0.05) from session 1–5 (STHA), and 1–10 (MTHA) during ODHA and TDHA. These physiological and perceptual adaptations were greater following session 10 (MTHA) compared to session 5 (STHA) (*P* < 0.05). Adaptations did not differ between HA groups (all *P* > 0.05), but larger magnitudes in adaptations were observed compared to both TEMP interventions (*P* < 0.05) (Table 2).

There were no differences (*P* > 0.05) between groups for exercise time, intensity or work completed during the HA or TEMP sessions. However, as expected physiological strain (i.e., time >38.5°C and ∆*T*_re_) was larger (*P* < 0.05) during HA compared to TEMP (Table 3). Exercise time and work completed during exercise sessions were greater (*P* < 0.05) between sessions 1–10 (MTHA) and sessions 1–5 (STHA) for each group (Table 3).

**Table 3:** Mean ± SD exercise data for sessions 1–5 (short‐term) and 1–10 (medium‐term)

Group	ODHA (44.4 ± 1.2°C, 21.1 ± 2.4% RH)†, ‡		TDHA (44.3 ± 1.3°C, 22.2 ± 3.9% RH)†, ‡		ODTEMP (21.6 ± 1.1°C, 40.9 ± 4.2% RH)		TDTEMP (21.8 ± 1.0°C, 38.6 ± 4.7% RH)	
Session	1–5	1–10	1–5	1–10	1–5	1–10	1–5	1–10
Time (min)	300 ± 0	600 ± 0*, +	300 ± 0	600 ± 0*, +	300 ± 0	600 ± 0*, +	300 ± 0*	600 ± 0*, +
Total work (kJ)	2378 ± 280	4838 ± 573*, +	2338 ± 211	4751 ± 374*, +	2419 ± 199	4834 ± 405*, +	2361 ± 254*	4778 ± 440*, +
Mean power (W·kg^−1^)	1.7 ± 0.1	1.7 ± 0.2	1.7 ± 0.1	1.7 ± 0.1	1.7 ± 0.1	1.7 ± 0.1	1.6 ± 0.2	1.6 ± 0.2
Mean power (% PPO)	49 ± 4	49 ± 3	47 ± 5	47 ± 5	47 ± 5	47 ± 5	46 ± 6	46 ± 6
*T*_repeak_ (°C)	38.45 ± 0.33	38.39 ± 0.36	38.52 ± 0.24	38.44 ± 0.21	38.39 ± 0.32	38.27 ± 0.26	38.15 ± 0.22	38.16 ± 0.30
∆*T*_re_ (°C)	1.29 ± 0.21†, ‡, ∂	1.52 ± 0.23*, †, ‡, +, ∂	1.48 ± 0.22†, ‡, ∂	1.68 ± 0.28*, †, ‡, +, ∂	0.70 ± 0.17	0.69 ± 0.18	0.78 ± 0.17	0.90 ± 0.19
Time >38.5°C (min)	62.8 ± 61.2†, ‡, ∂	118.6 ± 118.1*, †, ‡, +, ∂	50.2 ± 30.3†, ‡, ∂	90.9 ± 49.5*, †, ‡, +, ∂	21.3 ± 24.5	23.5 ± 17.0	21.5 ± 24.7	23.5 ± 18.8
HR_peak_ (b·min^−1^)	167 ± 15	163 ± 13	163 ± 11	155 ± 11+	169 ± 19	167 ± 17	177 ± 9	163 ± 6+
Sweat loss (mL)	980 ± 287	1513 ± 504*, †, ‡, +	1146 ± 429†, ‡	1545 ± 375*, †, ‡, +	655 ± 95	736 ± 139	616 ± 150	733 ± 150
∆PV (%)	−7.9 ± 4.0	−4.4 ± 2.8+	−9.4 ± 5.5	−4.6 ± 3.3+	−3.2 ± 2.6	−2.3 ± 1.1	−2.2 ± 1.4	−1.8 ± 1.1
RPE_peak_	15 ± 1†	13 ± 1*, †, ‡, +	15 ± 1†	14 ± 1*, †, +	17 ± 1	16 ± 2*	16±1	16 ± 2
TSS_peak_	6.8 ± 0.4†, ‡	6.1 ± 0.6*, +	7.0 ± 0.4†, ‡	6.1 ± 0.4*, +	5.8 ± 0.5	5.9 ± 0.7	6.2 ± 0.7	6.1 ± 0.5
TC_peak_	4 ± 1	2 ± 1*, +	4 ± 1	3 ± 1*, +	3 ± 0	3 ± 1	3 ± 0	3 ± 0

ODHA, once‐daily heat acclimation; TDHA, twice‐daily heat acclimation; ODTEMP, once‐daily temperate exercise; TDTEMP, twice‐daily temperate exercise; *T*_re_, rectal temperature; HR, heart rate; PV, plasma volume; ∆, change; RPE, rating of perceived exertion; TSS, thermal sensation; TC, thermal comfort.

* Represents a (*P* < 0.05) within‐group difference.

† Represents a (*P* < 0.05) between‐group difference with ODTEMP.

‡ Represents a (*P* < 0.05) between‐group difference with TDTEMP.

+ Represents a significant difference (*P* < 0.05) between 1–5 and 1–10 adaptations.

∂ Represents a significant (*P* < 0.05) between‐intervention difference (e.g., HA vs. TEMP).

Postintervention HASTs demonstrated reductions in sweat set point, HR_peak_ and TC_peak_, and improvements in sweat gain and WBSL (*P* < 0.05) for ODHA and TDHA groups, with greater improvements compared to TEMP (*P* < 0.05); yet no differences were found between HA protocols (*P* > 0.05) (Table 2).

### Exercise tolerance

#### Determinants of aerobic performance – GXT

A main effect was found for power output at LT and $\dot{V}O_{2\mathrm{peak}}$ (*P* < 0.05), with a greater (*P* < 0.05) improvement following HA (ODHA and TDHA), compared to TEMP (ODTEMP and TDTEMP; Table 4). No Time*Condition*Frequency interaction (*P* > 0.05) was found for any GXT data. No improvements (*P* > 0.05) were found in PPO or GME.

**Table 4:** Mean ± SD changes (∆) in exercise tolerance (determinants of aerobic performance and aerobic performance)

Group	ODHA			TDHA			ODTEMP			TDTEMP		
	Pre	Post	∆ (% ∆)	Pre	Post	∆ (% ∆)	Pre	Post	∆ (% ∆)	Pre	Post	∆ (% ∆)
Determinants of aerobic performance												
Power at LT (W)	159 ± 20	166 ± 26	+7 ± 10∂ (+3.4 ± 4.9)	163 ± 30	170 ± 28	+7 ± 8∂ (+4.6 ± 5.4)	157 ± 21	160 ± 23	+3 ± 5 (+1.9 ± 2.8)	159 ± 17	160 ± 13	+1 ± 6 (+1.1 ± 4.1)
GME (%)	19.9 ± 1.0	21.0 ± 2.0	+1.0 ± 2.2	20.5 ± 1.7	20.8 ± 1.4	+0.2 ± 1.6	19.3 ± 1.7	19.2 ± 1.6	−0.1 ± 1.5	19.7 ± 1.9	19.7 ± 2.0	+0.1 ± 1.2
$\dot{V}O_{2\mathrm{peak}}$ (L·min^−1^)	3.76 ± 0.46	3.95 ± 0.52	+0.18 ± 0.12∂ (+4.6 ± 3.1)	3.74 ± 0.50	3.89 ± 0.45	+0.13 ± 0.09∂ (+3.7 ± 2.8)	3.73 ± 0.43	3.83 ± 0.45	+0.10 ± 0.09 (+2.6 ± 2.5)	3.69 ± 0.34	3.73 ± 0.31	+0.05 ± 0.07 (+1.4 ± 2.0)
PPO (W)	291 ± 39	304 ± 48	+13 ± 18 (+4.2 ± 5.7)	296 ± 50	308 ± 46	+11 ± 8 (+3.9 ± 3.3)	288 ± 27	291 ± 31	+3 ± 14 (+1.6 ± 4.1)	287 ± 18	296 ± 18	+6 ± 11 (+2.3 ± 4.1)
Aerobic performance												
TTTF_TEMP_ (s)	519 ± 151	588 ± 153	+68 ± 11 (+14 ± 4)	553 ± 74	631 ± 82	+78 ± 47 (+14 ± 9)	510 ± 102	553 ± 106	+42 ± 51 (+9 ± 10)	532 ± 116	579 ± 161	+47 ± 62 (+8 ± 18)
TTTF_HOT_ (s)	412 ± 111	516 ± 140*	+104 ± 31 (+25 ± 4)*	450 ± 85	558 ± 117*	+109 ± 57 (+24 ± 11)*	416 ± 131	435 ± 149	+19 ± 58 (+5 ± 14)	430 ± 91	444 ± 97	+15 ± 77 (+5 ± 17)

ODHA, once‐daily heat acclimation; TDHA, twice‐daily heat acclimation; ODTEMP, once‐daily temperate exercise; TDTEMP, twice‐daily temperate exercise; LT, lactate threshold; GME, gross mechanical efficiency; $\dot{V}O_{2\mathrm{peak}}$, peak oxygen uptake; PPO, peak power output; TTTF_TEMP_, time to task failure in temperate condition; TTTF_HOT_, time to task failure in heat stress.

* Represents a significant (*P* < 0.05) within‐group difference.

∂ Represents a significant (*P* < 0.05) between‐intervention difference (e.g., HA vs. TEMP).

#### Aerobic performance ‐ TTTF

Preintervention TTTF_HOT_ was shorter (all *P* < 0.001) compared to TTTF_TEMP_ for all groups, with no between‐group differences (*P* > 0.05).

TTTF_HOT_ improved (*P* < 0.001) following ODHA and TDHA, but not ODTEMP or TDTEMP (*P* > 0.05), whereas TTTF_TEMP_ did not improve (*P* > 0.05) following any intervention (Table 4). Following TDHA and ODHA only, *T*_re_ and HR were lower at isotime (*P* < 0.05) during TTTF_HOT_ and TTTF_TEMP_ (Table 5).

**Table 5:** Mean ± SD changes (∆) in physiological measures compared to preintervention time to task failure in temperate (TTTF_TEMP_) and hot conditions (TTTF_HOT_)

Group	ODHA	TDHA	ODTEMP	TDTEMP
TTTF_TEMP_				
∆*T*_re_ (°C)	−0.21 ± 0.12*	−0.29 ± 0.24*	−0.14 ± 0.16	−0.14 ± 0.28
∆HR(b·min^−1^)	−6 ± 8*	−6 ± 4*	+1 ± 7	−3 ± 10
$\Delta \dot{V}O_{2}$ (L·min^−1^)	−0.02 ± 0.21	0.00 ± 0.17	−0.03 ± 0.32	−0.02 ± 0.28
∆RER	−0.08 ± 0.15	−0.01 ± 0.10	−0.06 ± 0.05	−0.07 ± 0.08
$\Delta {\dot{H}}_{\mathrm{prod}}$ (W)	−26 ± 73	−28 ± 72	+36 ± 127	+12 ± 170
$\Delta {\dot{V}}_{E}$ (L·min^−1^)	−8.4 ± 20.1	+5.2 ± 16.0	+8.1 ± 16.3	−6.2 ± 21.4
TTTF_HOT_				
∆*T*_re_ (°C)	−0.26 ± 0.26*	−0.26 ± 0.27*	−0.14 ± 0.28	−0.16 ± 0.33
∆HR(b·min^−1^)	−6 ± 5*	−8 ± 6*	0 ± 6	−3 ± 7
$\Delta \dot{V}O_{2}$ (L·min^−1^)	+0.01 ± 0.29	−0.09 ± 0.20	+0.03 ± 0.22	−0.05 ± 0.12
∆RER	−0.01 ± 0.08	+0.02 ± 0.06	−0.04 ± 0.10	−0.07 ± 0.08
$\Delta {\dot{H}}_{\mathrm{prod}}$ (W)	−17 ± 104	−11 ± 99	+16 ± 179	+20 ± 209
$\Delta {\dot{V}}_{E}$ (L·min^−1^)	−5.5 ± 20.4	−2.3 ± 16.0	+5.7 ± 20.7	+1.9 ± 16.0

ODHA, once‐daily heat acclimation; TDHA, twice‐daily heat acclimation; ODTEMP, once‐daily temperate exercise; TDTEMP, twice‐daily temperate exercise; *T*_re_, rectal temperature; HR, heart rate; $\dot{V}O_{2\mathrm{peak}}$, oxygen uptake; RER, respiratory exchange ratio; ${\dot{H}}_{\mathrm{prod}}$, metabolic heat production; ${\dot{V}}_{E}$, ventilation; ∆, change; TTTF_TEMP_, time to task failure in temperate condition, and TTTF_HOT_, time to task failure in heat stress.

* Represents a significant (*P* < 0.05) within‐group difference.

### Biomarkers

**Figure 2:**
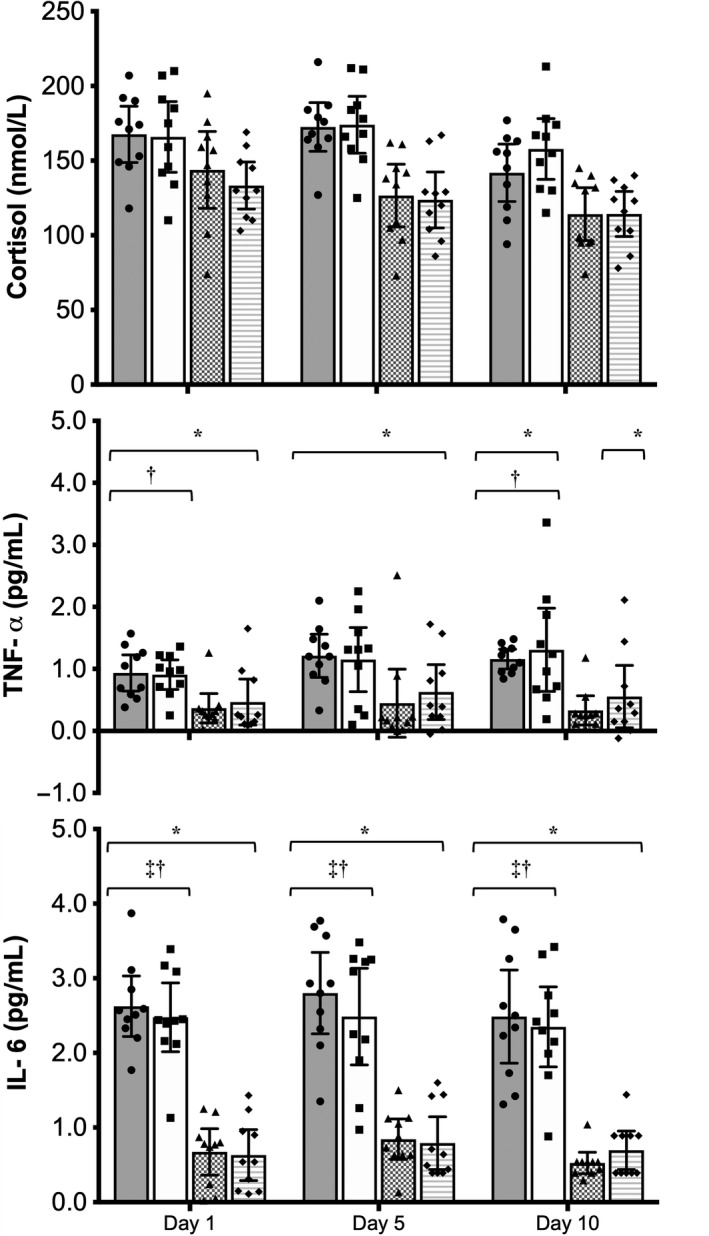
Mean ± SD changes in cortisol, TNF‐*α* and IL‐6 for session 1, 5, 10. *represents a significant (*P* < 0.05) within‐group difference pre‐ to postsession. ^†^represents a significant (*P* < 0.05) between‐group difference with ODTEMP. ^‡^represents a significant (*P* < 0.05) between‐group difference with TDTEMP. Shapes denote individual participants within‐group.

Increased plasma [IL‐6], [TNF‐*α*], and [cortisol] (*P* < 0.05) were observed from pre‐ to postsessions 1, 5, and 10 during both HA and TEMP protocols (Fig. 2). Inflammatory and stress responses were greater for HA compared to TEMP with larger mean: ∆IL‐6 values following sessions 1, 5, and 10 (*P* < 0.001); ∆TNF‐*α* following sessions 1 and 10, but not 5 when comparing HA to ODTEMP only (*P* < 0.05); and ∆cortisol following session 5 for ODHA versus TEMP, and following sessions 5 and 10 for TDHA versus TEMP (Fig. 2). No differences in inflammatory or stress responses were observed between the HA protocols at any time‐point (*P* < 0.05). Interestingly, there was no evidence of chronic effects over the course of HA or TEMP (*P* > 0.05); however, there was a trend (*P* < 0.10) for the ∆IL‐6 and ∆cortisol to be lower and ∆TNF‐*α* to be higher for session 10 when compared to the other sessions for ODHA and TDHA only.

## Discussion

In agreement with our hypothesis, ODHA and TDHA induced comparable heat adaptations to one another, thus demonstrating an improved heat acclimation state compared to ODTEMP and TDTEMP. Improvements in power at LT and $\dot{V}O_{2\mathrm{peak}}$ were found following HA, in addition to both ODHA and TDHA enhancing performance (TTTF) in hot, but not temperate conditions, an improvement that was not observed by either TEMP group. Inflammatory responses increased acutely following single sessions in all groups, with larger responses during HA versus TEMP. However, contrary to out hypothesis, no difference was observed between ODHA and TDHA groups. These data highlight that nonconsecutive TDHA presents no difference to ODHA, inducing similar heat adaptation and improvements in exercise tolerance during heat stress, without compromising immune status. These findings suggest the dose of HA (e.g., matched weekly exposure and intensity) is most important for the mechanisms which underpin adaptation, as opposed to the structure of HA (e.g., frequency [once‐ or twice‐daily] and timing [morning or afternoon]).

### Heat adaptations

HA efficacy was confirmed by the acquisition of key physiological heat adaptations including reductions in resting *T*_re_ (−0.3°C) and HR (−10 b·min^−1^), [Na^+^] retention (−14 to −27 mmol·L^−1^) and, increased WBSL (+398 to +533 mL) and PV expansion (+8.5 to +10.1) (Table 2). Proportional improvements were also observed following just 5 sessions (i.e., STHA). Reductions in RPE (−2) and TSS (−0.7 to −0.9), and an improved TC (−1) also demonstrate positive perceptual improvements following 10 sessions of both ODHA and TDHA. Collectively, these adaptations are in line with a recent meta‐analysis on HA (Tyler et al. 2016) and, while direct comparisons across studies are difficult due to differences in HA exercise protocols, MTHA studies (i.e., once‐daily) do report equivalent magnitudes of adaptation to the present study [e.g., resting *T*_re_: −0.17°C, and HR: −5 b·min^−1^, [Na^+^] retention: −22 mmol·L^−1^, WBSL: +29% and PV expansion: +4.3% (Tyler et al. 2016)]. Moreover, ODHA and TDHA induced adaptation superior to our predefined analytical limits (∆*T*_re_  > 0.20°C, ∆HR >5 b·min^−1^, ∆WBSL >200 mL, ∆PV >5% and >1 in perceptual scales [RPE, TSS, and TC] (Willmott et al. 2017)) highlighting meaningful heat adaptations, a critical factor when assessing intervention strategies.

Both HA strategies improved heat acclimation state, as indicated by a lower sweat set point (−0.22°C) and a larger sweat gain (+0.14 to +0.15 g·sec^−1^·°C^−1^) during the postintervention HAST (Table 2).While no reductions in ∆*T*_re_, *T*_repeak_, RPE_peak_, or TSS_peak_ occurred following HA, this can be explained by the represcription of exercise intensities, thus, controlling for ${\dot{H}}_{\mathrm{prod}}$ postintervention and providing confidence in our adaptations. The unchanged *T*_re_ but larger WBSL (both +35%) shows thermosensitivity is enhanced via increased sweat gain for ODHA (+48%) and TDHA (+49%). Though these changes are superior to the meta‐analysis findings (+25% (Tyler et al. 2016)) and TEM (0.09 g·sec^−1^·°C^−1^ (Willmott et al. 2015)) the authors accept that an esophageal core temperature and real‐time local sweat rate measurements would offer superior assessment of these data given a more rapid response in comparison to our rectal thermistor (Taylor et al. 2014). Parallel reductions in resting *T*_re_ and sweat set point, following ODHA (−0.28 and −0.22°C) and TDHA (−0.28 and −0.22°C), respectively, agree with meta‐analysis findings (−0.28 (Tyler et al. 2016)) and are larger than the TEM (0.21°C (Willmott et al. 2017)). MTHA (e.g., 10 sessions) induced greater magnitudes of physiological and perceptual heat adaptation compared to STHA (e.g., 5 sessions [Table 2 and 3]). Though not in agreement with all experimental data (Gibson et al. 2015a,b), these findings agree with consensus recommendations that longer‐term HA (e.g., ≥10 days) is preferable to induce greater physiological heat adaptations (Racinais et al. 2015; Periard et al. 2016) achieved in this study through the maintained physiological strain imposed using isothermic prescription (Taylor 2014). These data provide supporting evidence that medium‐ to long‐term HA could be prescribed immediately before, or potentially several weeks before major athletic competition or military deployment in heat stress (Daanen et al. 2017) to induce greater initial adaptations, as opposed to solely implementing STHA during the final training microcycle. This notion, alongside the decay of these aforementioned adaptations (Daanen et al. 2017), should be experimentally examined as this strategy would allow alternate approaches (e.g., intermittent “top up” exposures in the days preceding exposure) to be implemented to maintain the enhanced heat acclimation state (Casadio et al. 2016).

Seminal work by Lind and Bass (Lind and Bass 1963) demonstrated the benefits of continuous, once‐daily HA (i.e., 100‐min sessions), as opposed to longer and shorter intermittent times (e.g., twice‐daily, 2 × 50 min), which contributed to duration recommendations for optimal heat adaptations (Racinais et al. 2015). Our data indicate no advantage but more importantly, no disadvantage of nonconsecutive TDHA over consecutive ODHA, agreeing with our previous STHA investigation (Willmott et al. 2016). Further to this, as outlined above, these observations are true even when the session duration is 60 min (this study), as opposed to 90–100 min which has been previously described as preferable (Racinais et al. 2015). Our novel findings are in contrast to others which have not demonstrated efficacy of TDHA (Gill and Sleivert 2001), but may be explained by a) the use of an isothermic model, b) the matching of exercise‐heat dose (e.g., duration, intensity and total number of exposures) to induce equivalent heat adaptations and improved exercise tolerance, and/or c) more significant heat strain, that is maximizing time spent at the targeted *T*_re_. This nonconsecutive twice‐daily structure is likely to be appealing to coaches and practitioners with upcoming competitions in challenging, hot conditions (e.g., Tokyo 2020 Olympic and Paralympic Games) for whom scheduling HA around sport‐specific training, competition tapering, rest and travel are challenging. This study is the first to demonstrate equivalent heat adaptations following both TDHA and ODHA, with greater adaptations for longer interventions (i.e., 5‐ vs. 10 days) suggesting the dose of HA (i.e., attaining key physiological responses to a greater extent) is the primary factor that underpins adaptation.

### Exercise tolerance

#### Determinants of aerobic performance

Our study provides a holistic overview of the changes in exercise tolerance following nonconsecutive TDHA, in comparison to consecutive ODHA and matched TEMP interventions. $\dot{V}O_{2\mathrm{peak}}$ improved following HA (ODHA: +4.6%; TDHA: +3.7%), with this change greater than TEMP changes (ODTEMP: +2.6%; TDTEMP: +1.4%). This is likely due to hypervolemia following HA and potential increments in cardiac output (Lorenzo et al. 2010); however, it must be acknowledged that participants were not elite athletes whom as a cohort may be less responsive to this mechanism (Nybo and Lundby 2015a). Nonetheless, previous studies report ergogenic benefits of HA on $\dot{V}O_{2\mathrm{peak}}$ and PPO in temperate conditions (Sawka et al. 1985; Takeno et al. 2001; Lorenzo et al. 2010; Fujii et al. 2012, 2015; Rendell et al. 2017) while others present no changes (Karlsen et al. 2015; Keiser et al. 2015; Neal et al. 2015). Power at LT also improved significantly following HA, in agreement with previous findings (Lorenzo et al. 2010; Petersen et al. 2010; Chalmers et al. 2014, 2016; Neal et al. 2015; Kelly et al. 2016; Rendell et al. 2017; James et al. 2018); however, improvements following ODHA (+7 ± 10 W) and TDHA (+7 ± 8 W) were of a lower magnitude than those reported in well‐trained cyclists in 13°C (+12–15 W (Lorenzo et al. 2010)) and 22°C (+16 W [(Neal et al. 2015)] and +15 W [(Rendell et al. 2017)]). Furthermore, GME did not change following interventions, in agreement with previous LTHA (Karlsen et al. 2015). While the ergogenic benefits of HA remain disputed, potentially as a result of insufficient potentiating stimuli or inter‐individual differences (Corbett et al. 2018), our data are the first to demonstrate that implementing a nonconsecutive twice‐daily intervention does not induce differential ergogenic effects to that of a matched dose once‐daily protocol, for the determinants of aerobic performance (e.g., $\dot{V}O_{2\mathrm{peak}}$ and power at LT) in temperate conditions.

#### Aerobic performance – time to task failure determinants of aerobic performance

TTTF_HOT_ improved following ODHA (+25%) and TDHA (+24%), but not ODTEMP and TDTEMP (both +5%), agreeing with previous reports following MTHA (+67% [(Nielsen et al. 1993)], +17% [(Nielsen et al. 1997)], and +24% [(Daanen et al. 2011)]), which appear to exceed STHA (+14% [(Garrett et al. 2009)] and +7% [(Chen et al. 2013)]) likely due to greater physiological adaptation. Evidence for TTTF_HOT_ improvements likely reflecting the magnitude HA adaptations (e.g., PV expansion improving cardiac output (Lorenzo et al. 2010; Nielsen et al. 1993), leading to increased $\dot{V}O_{2\mathrm{peak}}$ and power at LT, resulting in a lessened physiological strain (Periard et al. 2016), is indicated by a lower mean *T*_re_ (−0.26°C) and HR (−8 b·min^−1^) at isotime (Table 5). Consequently, nonconsecutive TDHA appears equally effective as ODHA for improving aerobic performance (e.g., extending exercise tolerance time) in subelite athletes within the severe‐intensity domain under heat stress. It is likely adaptations contributed to the improved TTTF_TEMP_ following ODHA and TDHA (both +14%). However, these data describe that HA (irrespective of once‐ or twice‐daily frequency) provided only moderate ergogenic benefits for performance in temperate conditions, opposing significant time trial improvements in 13°C (Lorenzo et al. 2010) and 22°C (Rendell et al. 2017) but agreeing with recent studies which suggest these physiological constructs are not limitng (Karlsen et al. 2015; Keiser et al. 2015; Neal et al. 2015). Nonetheless, this is the first study to collectively assess TTTF in both hot and temperate conditions which while demonstrating some inter‐individual differences (Fig. 2), describes ODHA and TDHA as providing ergogenic benefits for enhanced performance in hot conditions with data in temperate conditions encouraging, albeit not unequivocal (Minson and Cotter 2015; Nybo and Lundby 2015b).

### Inflammatory and stress responses

**Figure 3:**
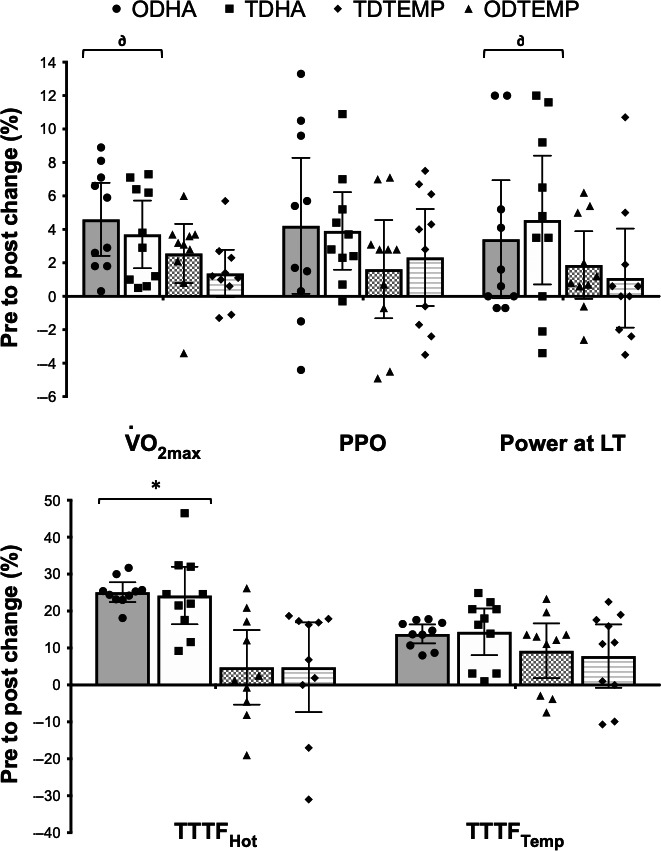
Mean ± SD changes in the determinants of aerobic performance and aerobic performance in hot and temperate conditions. *represents a significant (*P* < 0.05) within‐group difference pre‐ to postsession. ^**∂**^represents a significant (*P* < 0.05) between‐group difference with (HA versus TEMP). Shapes denote individual participants within‐group.

Agreeing with previous literature, larger ∆IL‐6 and ∆TNF‐*α* were observed during HA compared to TEMP (Fig. 3) (Starkie et al. 2005; Peake et al. 2007; Hailes et al. 2011; Kuennen et al. 2011; Barberio et al. 2015; Guy et al. 2016; Lee et al. 2018). The larger responses observed for ∆cortisol for sessions 5 and 10, but not 1 (Armstrong et al. 1990; Hargreaves et al. 1996; Brenner et al. 1997; Starkie et al. 2005; Peake et al. 2007; Cooper et al. 2010; Hosick et al. 2010) during HA are a response to increased physiological strain due to the heat stress for the same absolute exercise intensity (e.g., higher *T*_re_, ∆*T*_re_ and HR) (Starkie et al. 2005). Our changes in IL‐6 (+55%), TNF‐*α* (+45%) and cortisol (+34%) during HA were comparable in ODHA and TDHA, and are less than, or comparable to, responses published elsewhere (IL‐6: +20–2000%, TNF‐*α*: +15–65%, and cortisol: +20–70%) (Armstrong et al. 1990; Brenner et al. 1997; Starkie et al. 2005; Peake et al. 2007; Hosick et al. 2010; Hailes et al. 2011; Kuennen et al. 2011; Barberio et al. 2015; Guy et al. 2016; Costello et al. 2018). Our findings also agree with reported transient ∆IL‐6 during MTHA (Guy et al. 2016; Costello et al. 2018) alongside induced heat adaptations (Rendell et al. 2017) and no evidence of chronic inflammatory effects or signs of exaggerated ∆TNF‐*α* (e.g., possible endotoxemia) (Guy et al. 2016). The absence of augmented ∆cortisol as HA progresses, conforms to previous literature describing the sensitivity of this biomarker to various stressors (Armstrong et al. 1990; Sunderland et al. 2008; Watkins et al. 2008; Garrett et al. 2009; Costello et al. 2018). In summary, our data indicate no chronic inflammatory effects or stress responses during ODHA and, for the first time during nonconsecutive TDHA, which is likely due to the equivalent acquisition in physiological heat adaptation. These novel data provide confidence that our TDHA protocol did not induce unexpected inflammatory or stress responses which could compromise immune status in subsequent heat exposures to any greater extent than ODHA. This further strengthens the argument for TDHA when ODHA is impractical.

### Application

The similarity of the responses to nonconsecutive TDHA and ODHA, may be of particular interest to sporting and occupational organizations that require heat adaptations to lessen the physiological strain and HRI risk, and improve exercise performance in heat stress. Nonconsecutive TDHA provides an alternate and flexible strategy, providing the potential to half the number of interrupted training days, thus maximizing an individual's time to complete specific (e.g., nonheat) training or rest/recover without compromising the magnitude of adaptation. Logistically, the nonconsecutive TDHA is appealing given the cost and time associated with athletes or workers travelling to specialist heat training facilities in cool climates, may be reduced if multiple heat sessions can be completed on 1 day. The transient nature of heat adaptations requires STHA during crucial preparation periods, where training is predominantly sport‐specific with volume often adjusted to optimize recovery, thus resulting in training that opposes targeted physiological adaptations. It is unsurprising therefore, that repeated steady‐state exercise during consecutive day HA, do not appear to be widely embraced by competitive athletes (Périard et al. 2017). Prescribing TDHA and specifically afternoon sessions, may also increase HA efficiency as time spent at the desired isothermic *T*_re_ of >38.5°C was extended during afternoon compared to morning sessions (+14 vs. +6 min); yet ∆*T*_re_ were lower (+1.3°C vs. +1.6°C), thus requiring less exercise time to reach target temperatures due to circadian rhythm and higher resting *T*_re_. Ultimately, shorter duration HA (~60 min) that provides sufficient physiological strain to evoke meaningful phenotypic adaptations irrespective of daily frequency and consecutive scheduling is desirable, with nonconsecutive TDHA providing greater flexibility than a consecutive day protocol.

### Limitations and future direction

Despite our biomarker data indicating TDHA do not induce excessive inflammatory/immune responses, our mechanistic insights are limited due to the number and timing of blood sampling. Collecting additional biomarker measures and across more time‐points during the recovery phase (e.g., 1–24 h) would provide further insight into the inflammatory responses and potential maladaptive influences on the magnitude and kinetics of heat adaptation. An extension of this work would also examine intracellular heat shock proteins (Kuennen et al. 2011) and the relevant gene transcripts (Gibson et al. 2015a) to elucidate the impact of TDHA versus ODHA on attaining thermotolerance (Kuennen et al. 2011), and potential benefits across environmental stressors (Gibson et al. 2015c, 2017a). We also highlight a need to investigate the precise effect of consecutive and nonconsecutive TDHA in females, who experience different thermoregulatory adaptation kinetics to males (Mee et al. 2015). Moreover, the effect of HA duration should be considered (e.g., 60‐ vs. 90/100‐min sessions) given an extended heat dose may impact the kinetics and magnitude of both adaptation and the inflammatory responses. A paucity of data still exists to effectively characterize the rate of heat decay and re‐induction of HA at a physiological and molecular level, which is critical for the implementation of all HA protocols including TDHA. Finally, we highlight the need for investigations regarding the feasibility and appropriateness of HA and other concurrent training (e.g., interval or competition specific intensity sessions) for elite athletes.

## Conclusion

This is the first study to investigate the efficacy of nonconsecutive twice‐daily HA compared to daily HA for adaptations, biomarkers, and exercise tolerance. Greater heat adaptations were induced by both once‐ and twice‐daily HA protocols, compared with equivalent temperate exercise, without adverse effects on inflammatory or stress responses. Exercise tolerance in heat stress was improved following both HA protocols, yet no effect was found for matched‐volume TEMP, nor were improvements found for exercise tolerance in temperate conditions for all interventions. The concomitant increase in power at LT and $\dot{V}O_{2\mathrm{peak}}$ following HA, reaffirms the erogenicity of HA on aerobic performance within heat stress, although our data do not provide supportive evidence for HA to enhance aerobic performance in temperate conditions.

## Conflict of Interest

The authors confirm there is no conflict of interest.
